# Codelivery of anti-CD47 antibody and chlorin e6 using a dual pH-sensitive nanodrug for photodynamic immunotherapy of osteosarcoma

**DOI:** 10.32604/or.2023.030767

**Published:** 2024-03-20

**Authors:** JIJIE XIAO, HONG XIAO, YUJUN CAI, JIANWEI LIAO, JUE LIU, LIN YAO, SHAOLIN LI

**Affiliations:** 1 Department of Radiology, The Fifth Affiliated Hospital of Sun Yat-Sen University, Zhuhai, 510900, China; 2 Department of Ultrasound, The Third Affiliated Hospital of Sun Yat-Sen University, Guangzhou, 528405, China; 3 PCFM Lab of Ministry of Education, School of Materials Science and Engineering, Guangzhou, 510275, China

**Keywords:** Immunotherapy, Osteosarcoma, Nanodrug, Photodynamic therapy, CD47

## Abstract

Osteosarcoma is a malignant tumor originating from bone tissue that progresses rapidly and has a poor patient prognosis. Immunotherapy has shown great potential in the treatment of osteosarcoma. However, the immunosuppressive microenvironment severely limits the efficacy of osteosarcoma treatment. The dual pH-sensitive nanocarrier has emerged as an effective antitumor drug delivery system that can selectively release drugs into the acidic tumor microenvironment. Here, we prepared a dual pH-sensitive nanocarrier, loaded with the photosensitizer Chlorin e6 (Ce6) and CD47 monoclonal antibodies (aCD47), to deliver synergistic photodynamic and immunotherapy of osteosarcoma. On laser irradiation, Ce6 can generate reactive oxygen species (ROS) to kill cancer cells directly and induces immunogenic tumor cell death (ICD), which further facilitates the dendritic cell maturation induced by blockade of CD47 by aCD47. Moreover, both calreticulin released during ICD and CD47 blockade can accelerate phagocytosis of tumor cells by macrophages, promote antigen presentation, and eventually induce T lymphocyte-mediated antitumor immunity. Overall, the dual pH-sensitive nanodrug loaded with Ce6 and aCD47 showed excellent immune-activating and anti-tumor effects in osteosarcoma, which may lay the theoretical foundation for a novel combination model of osteosarcoma treatment.

## Introduction

Osteosarcoma is a primary malignant tumor that originates from mesenchymal tissue, which is well developed in the metaphysis of long bones, is one of the most common cancers in children and adolescents [1,2]. Currently, osteosarcoma accounts for approximately 2.4% of malignancies in children and adolescents worldwide [3]. Osteosarcoma is characterized by the presence of spindle-shaped stromal cells and osteoid [4]. And the typical symptoms of patients are local swelling, pain and functional limitation. The overall prognosis for patients with osteosarcoma is poor, with approximately 10%–20% of patients with osteosarcoma having metastases (mainly lung metastases) at the time of diagnosis [5]. The 5-year survival rate for patients with chemotherapy-resistant or pulmonary metastases is less than 20% [6]. Current standard treatment for osteosarcoma includes primary tumor resection, combined with adjuvant or neoadjuvant chemotherapy [7]. Unfortunately, patient survival rates remain dismal, and the long-term use of intravenous chemotherapy always leads to severe side effects, which can include death [8]. Therefore, more effective therapy options with fewer side effects are urgently needed.

Immune checkpoints are protein molecules that are expressed on the surface of immune cells and regulate the degree of immune activation, and their abnormal expression is a key mechanism for tumor development [9,10]. Immune checkpoint blockade therapy is a systemic approach to enhance the efficacy of systemic anti-tumor therapy by using immune checkpoint inhibitors to target and bind to these receptors, thereby blocking the signaling pathways that inhibit the anti-tumor immune response and enhancing the effective recognition of cancer cells by the immune system [11,12]. Blocking immune checkpoints has been applied in various clinical treatments, including for lung cancer and urothelial carcinoma [13–15]. CD47, a small molecule expressed on the surface of tumor cells, functions as a “don’t eat me” mark by binding to signal-regulatory protein alpha (SIRPα), an inhibitory receptor, on tumor-associated macrophages (TAMs) and dendritic cells (DCs) [16–19]. CD47 monoclonal antibody (aCD47) can prevent the binding of CD47 and SIRPα, thereby promoting tumor cell phagocytosis by TAMs and DCs [20,21]; however, many challenges remain to be overcome in application of aCD47 in osteosarcoma, including low immune response rates and systemic side effects.

As an emerging approach for cancer treatment, photodynamic therapy (PDT) has characteristics of high spatiotemporal selectivity and noninvasiveness [22,23]. In PDT, a photosensitizer exposed to light transfers energy to oxygen, which generates reactive oxygen species (ROS) that can directly induce tumor cell apoptosis and necrosis [24,25]. Further, many studies have demonstrated that PDT also causes immunogenic cell death (ICD) of cancer cells [26]. During ICD, calreticulin (CRT) is translocated to the cell membrane and ATP and high-mobility group box protein 1 (HMGB1) are released, which facilitates DC maturation to stimulate T cell-mediated ICD [27–30]. Furthermore, CRT evokes cancer cell phagocytosis by TAMs and DCs [31,32].

It is established that polymeric nanocarriers can accumulate in tumors via elevated permeability and retention (EPR) or active targeting effects, which can enhance their efficacy and reduce systemic side effects [33,34]. Moreover, nanocarriers can be designed to carry certain drugs and release them in specific sites [35–37].

In this research, we introduced a dual pH-sensitive polymeric nanocarrier to co-deliver aCD47 and the photosensitizer, chlorin e6 (Ce6), which has not been explicitly reported, to achieve combined PDT and immunotherapy. Due to the pH sensitivity of the system, aCD47 can be freed in the acidic tumor microenvironment (TME) to interfere with cancer cells via CD47. The surface charge of the nanocarrier changed to positive in the TME, which accelerated its cellular uptake. Further, when exposed to light, Ce6 both generated ROS to kill tumor cells and triggered tumor ICD, thereby converting a “cold tumor” into a “hot tumor”, and eventually activating anti-tumor immune responses. We also investigated whether the effect of Ce6 and aCD47 in cancer treatment was synergistic.

## Materials and Methods

### Preparation of an aCD47-decorated nanodrug, Ce6@PPC-aCD47

First, aCD47 was fused to the surface of Ce6@PPC micelles. Briefly, 5 ml of Ce6@PPC micelles (1 mg/ml) and 1 ml aCD47 (0.5 mg/ml) were mixed at 4°C for 24 h in dark. Then, free CD47 was eliminated by ultrafiltration. After concentrating the solution, free Ce6 was measured by ultraviolet-visible absorbance, and aCD47 was detected by enzyme-linked immunosorbent assay (ELISA). Ce6 and aCD47 loading content in micelles was calculated based on calibration curves.

### In vitro drug release study

The *in vitro* release of Ce6 and aCD47 from Ce6@PPC-aCD47 was measured by dialysis diffusion. Briefly, a dialysis bag (MWCO, 100 kDa) containing 2 ml Ce6@PPC-aCD47 was soaked in 8 ml release medium at different pH values and then incubated at 37°C with horizontal shaking. Dialysis buffer was removed and replaced with fresh buffer at pre-set intervals, and aCD47 and Ce6 detected. To further investigate CD47 antibody release, Ce6@PPC-aCD47 was incubated with Alexa fluor-488-conjugated IgG at pH 7.4, and free secondary antibody removed by ultrafiltration. Then, the pH value of samples was adjusted to 6.5 for 0, 6, 12, 24, or 48 h, and fluorescence intensity examined at 525 nm.

### Serum stability assay

To explore the stability of Ce6@PPC-aCD47, a 10% solution was generated by adding 0.1 ml Ce6@PPC-aCD47 into 0.9 ml PBS at 37°C. Then the diameters of the nanodrug were determined by dynamic lighting scattering (DLS).

### In vitro cytotoxicity experiments

Murine osteosarcoma cell line (K7M2) was cultured in Dulbecco’s modified eagle medium (DMEM, Gibco, Cat. No. 1859228) with 10% fetal bovine serum (FBS, Gibco) and the Cell Counting Kit 8 (CCK-8) assay used to measure the cytotoxicity of blank micelles, and the nanodrug with or without light irradiation. K7M2 cells were cultured for 24 h, treated with 100 μl fresh medium containing various concentrations of blank micelle and Ce6@PPC-aCD47. After incubation for 4 h and washing with PBS, cells were irradiated with or without 660 nm laser (1 W/cm^2^) for 1 min [38]. Then, cells were incubated for another 24 h, followed by addition of CCK-8 reagent (Beyotime, China), and incubation for 1 h before measurement of cell viability.

### Cell uptake of nanodrug in vitro

K7M2 cells were cultured in 35 mm confocal dishes for 24 h, then incubated with NR@PPC-aCD47 at pH 7.4 or 6.5 for 4 h, washed with PBS, and fixed in 4% paraformaldehyde (PFA) for 15 min. Nuclei were stained with DAPI. A confocal microscope was used for observation. For flow cytometric analysis, K7M2 cells were cultured for 24 h, then incubated with NR@PPC-aCD47 pretreated for 4 h at pH 7.4 or 6.5 for different periods of time. Finally, cells were collected for flow cytometry analysis.

### Cell apoptosis analysis in vitro

K7M2 cells were cultured for 24 h and treated with PBS, free Ce6, or Ce6@PPC-aCD47 at pH 7.4 or 6.5, containing identical concentrations of Ce6. Four hours later, after washing and irradiation (660 nm laser for 1 min (1 W/cm^2^)), cells were stained with Annexin V-FITC/propidium iodide (Sigma-Aldrich; Merck KGgA).

### ROS generation in vitro

K7M2 cells were treated with PBS, free Ce6, or Ce6@PPC-aCD47 at pH 7.4 or 6.5 containing an identical concentration of Ce6. After 4 h incubation, cells were stained with 2′,7′-dichlorofluorescein diacetate (DCFH-DA; Beyotime, China) for 1 h and then irradiated using a 680 nm laser for 1 min (1 W/cm^2^). Cells were harvested for flow cytometry. For confocal observation, K7M2 cells were cultured in 35 mm confocal dishes and treated with PBS, free Ce6, or Ce6@PPC-aCD47 at pH 7.4 or 6.5 containing identical concentrations of Ce6. After 4 h incubation, cells were stained with DCFH-DA and Hoechst 33342, and then exposed to a laser (660 nm, 1 W/cm^2^) for 1 min.

### Detection of ICD in vitro

To detect CRT expression by flow cytometry, K7M2 cells were first treated with PBS, free Ce6, or Ce6@PPC-aCD47 at pH 7.4 or 6.5 containing the same concentration of Ce6. After 4 h, cells were irradiated using a 660 nm laser for 1 min at 37°C, stained with anti-CRT antibody (Cell Signaling Technology, USA) and analyzed by flow cytometry. For imaging, cells treated with PBS, free Ce6, or Ce6@PPC-aCD47 at pH 7.4 or 6.5 containing the same concentration of Ce6 were added into different dishes. After 4 h, cells were stained with DCFH-DA and Hoechst 33342 for 30 min at 37°C in darkness, then exposed to a 660 nm laser for 1 min (1 W/cm^2^). After fixing, cells were stained with anti-mouse CRT antibody, secondary antibody, and DAPI, then observed under a confocal microscope. To detect HMGB1 and ATP, cells were treated by PBS, free Ce6, or Ce6@PPC-aCD47 at pH 7.4 or 6.5 containing the same concentration of Ce6. After 4 h, cells were treated with a 660 nm laser for 1 min (1 W/cm^2^) and cultured overnight. HMGB1 and ATP were measured in culture supernatants by ELISA.

### In vitro bone marrow-derived dendritic cell (BMDC) maturation assay

BMDCs were generated from 8-week-old BALB/c mice. K7M2 cells were pretreated with PBS, Ce6@PPC, PPC-aCD47, and Ce6@PPC-aCD47 at pH 6.5, to mimic the acidic tumor microenvironment, and exposed to a 660 nm laser for 1 min (1 W/cm^2^). Subsequently, 106 BMDCs were co-cultured with 2 × 10^5^ pretreated K7M2 cells for 24 h. After staining with anti-CD11c-FITC, anti-CD80-PE, and anti-CD86-APC antibodies (eBioscience, Invitrogen, USA), the percentage of mature BMDCs was measured by flow cytometry (BD Biosciences, USA), and the data were analyzed using FlowJo software (TreeStar Inc., USA).

### Animal model

Male BALB/c mice (4–5 weeks old) were from Guangdong Medical Lab Animal Center, China. This study was approved by the Research Ethics Committee of the Fifth Affiliated Hospital of Sun Yat-Sen University (00045). All care of, and experiments on, animals were carried out under international animal experiment guidelines. Procedures were approved by our university. K7M2 xenografts were established by subcutaneous injection of 4T1 cells. Once tumors were 100 mm^3^, mice were injected intravenously (i.v.) with PBS, Ce6@PPC, PPC-aCD47, or Ce6@PPC-aCD47 every other day (aCD47, Ce6: 1.0, 0.75 mg kg^−1^). Then, 24 h after injection, tumors were irradiated using a 660 nm laser for 1 min (1 W/cm^2^). Tumor volume was calculated 0.5 × length × width 2. When tumor volumes reached approximately 2000 mm^3^, mice were euthanized and tumors collected for subsequent experiments. There were eight mice in each group.

### Hematoxylin and eosin (H&E) staining

The tumor tissues of each group were fixed by 4% PFA, dehydrated by gradient alcohol, and transparent by xylene. After paraffin embedding, the tissues were made into paraffin sections. The sections were dewaxed with xylene, hydrated with gradient alcohol, washed with distilled water, stained with hematoxylin (Solarbio, China; Cat. No. H8070), fractionated with 1% hydrochloric acid alcohol, returned to blue with 0.6% ammonia and stained with eosin. Subsequently, the sections were dehydrated by gradient alcohol, transparent, and sealed with neutral gum. Histopathological patterns were observed and photographed under the microscope (Nikon, Tokyo, Japan).

### TdT-mediated dUTP-biotin nick end labeling (TUNEL) staining

The paraffin sections were dewaxed with xylene, hydrated with gradient alcohol, and processed with proteinase K (Sigma) for 30 min at 37°C. After PBS washing, the sections were added with TdT enzyme and fluorescent labeling solution at 37°C for 1 h. After PBS washing, the sections were incubated with DAPI for 30 min. After sealing with anti-fluorescence quenching solution, the results were observed and photographed by a fluorescence microscopy (Olympus, Tokyo, Japan).

### In vivo fluorescence imaging

Ce6@PPC-aCD47 labeled with 1,1′-dioctadecyl-3,3,3′,3′-tetramethylindotricarbocyanine iodide (DiR) were i.v. injected into tumor-bearing mice (DiR, 1 mg/kg). Fluorescence images were obtained using a Carestream IS 4000 instrument and major organs and tumors were harvested 48 h post-administration for *ex vivo* imaging.

### Toxicity assessment

To assess nanodrug side effects, liver and renal function were evaluated using serum markers (total bilirubin (TBIL), aspartate transaminase (AST), and creatinine (Cr)). Heart, lung, liver, spleen, and kidney were collected for H&E staining.

### Immune response in vivo

To examine DC maturation *in vivo*, tumors were homogenized to obtain single-cell suspensions. After staining with anti-CD11c-FITC (eBioscience, Invitrogen, USA; Cat No. 11-0114-85), anti-CD80-PE (Bioscience, Cat No. 12-0801-85), and anti-CD86-APC (eBioscience, Cat No. 17-0862), proportions of mature DCs were detected by flow cytometry. Anti-CD3-PerCP-Cy5.5, anti-CD4-FITC, and anti-CD8-PE antibodies were used to analyze T cell subpopulations. Anti-CD11b-FITC (BD Pharmingen), anti-F4/80-APC (eBioscience), anti-CD206-PE (eBioscience), and anti-CD80-Cy5.5 (eBioscience) were used to analyze TAMs. Single-cell suspensions were also generated from spleens. Anti-CD3-PerP-Cy5.5 (eBioscience, Cat No. 45-0031), anti-CD8-PE (eBioscience, Cat No. 12-0081), anti-CD44-AF700, and anti-CD62L-FITC antibodies were used for CD8+ T cell analysis.

### Immunofluorescence and immunohistochemical (IHC) analysis

For immunofluorescence staining, tumor sections were incubated with primary and secondary antibodies, counterstained with DAPI (Sigma-Aldrich, Cat No. D8417), and observed. For IHC, sections were deparaffinized, incubated in 3% H_2_O_2_, soaked in citrate buffer at 95°C, blocked, incubated with primary antibodies, rinsed, stained using diaminobenzidine (DAB; Sigma, Cat No. D7304-1SET), and scanned with an optical microscope (Olympus CK30). Staining degree: no staining (0), light yellow (1), brown yellow (2), brown (3); The percentage of positive cells: <5% (0), 5%–25% (1), 26%–50% (2), >51% (3). The expression score (0–9) was obtained by multiplication of the two markers. 0–2 indicated low expression; 3–9 indicated high expression.

### ELISA

For cells, we collected cell supernatants from each group; for tissues, the tumor tissue was ground, and the ground fluid was collected. Levels of IFN-γ and TNF-α were detected by ELISA (Westang, Shanghai, China).

### Statistical analysis

All experiments were repeated three times independently. And the data are presented as mean ± standard deviation (SD). Statistical differences among two or more groups were analyzed by SPSS version 20.0 (SPSS, Chicago, IL, USA) with Student’s *t*-test or ANOVA (*p < 0.05, **p < 0.01, ***p < 0.001).

## Results

### Nanodrug preparation and characterization

**Figure 1:**
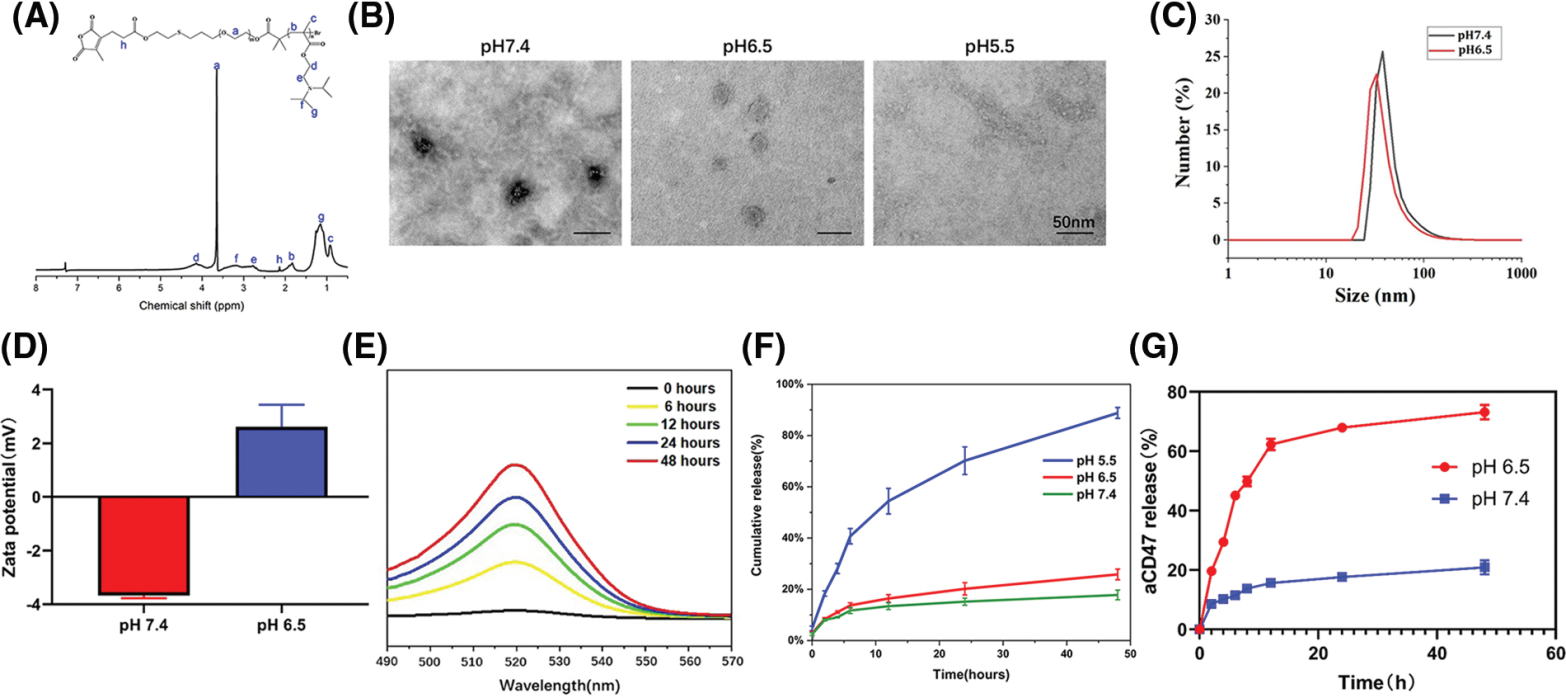
Characterization of Ce6@PPC-aCD47. (A) 1H NMR of CDM-PEG-PDPA in CDCl3. (B) TEM observation of Ce6@PPC-aCD47, scale bar = 50 nm. (C) Particle sizes of Ce6@PPC-aCD47 determined by dynamic light scattering. (D) Zata potential of Ce6@PPC-aCD47. (E) Fluorescence spectra of Alexa Fluor 488-labeled nanodrug in PBS at pH 6.5. (F) *In vitro* Ce6 release from Ce6@PPC-aCD47 at pH 6.5 and pH 7.4. (G) *In vitro* aCD47 release from Ce6@PPC-aCD47 at pH 6.5 and pH 7.4.

CDM-PEG-PDPA, CDM-PEG, and PDPA were synthesized through multistep reactions (Suppl. Fig. S1) [39] and 1H NMR analysis demonstrated successful syntheses of diblock copolymers (Fig. 1A). The Ce6-capped diblock copolymer was self-assembled to generate Ce6@PDPA-PEG-CDM nanomicelles (Ce6@PPC) with a PDPA core encapsulating Ce6. Finally, micelles were surface decorated with aCD47 via ammonolysis of the primary amino groups of the antibody with CDM, leading to successful synthesis of Ce6@PPC-aCD47. Ce6 and aCD47 loading content in the nanodrug were 4.3% and 2.6%, respectively. No obvious change in particle sized was detected in 10% FBS-containing PBS (Suppl. Fig. S2), suggesting good stability under normal physiological conditions. As shown in Fig. 1B, the nanodrug exhibited uniform spherical morphology at pH 7.4, with a core-shell structure under transmission electron microscope (TEM) observation. The nanodrug shell was less obvious at pH 6.5, owing to CDM cleavage-induced antibody detachment. In contrast, the nanodrug completely disintegrated at pH 5.5, with only a few fragments detected. The hydrodynamic diameter of Ce6@PPC-aCD47 decreased slightly at lower pH (33 *vs*. 40 nm), as a result of antibody release (Fig. 1C). In addition, owing to the negatively charged antibody, the zeta potential of Ce6@PPC-aCD47 was −3.67 ± 0.11 mV at pH 7.4 (Fig. 1D), which suggests that the particles will be stable in the blood circulation and accumulate in tumor tissue [37,40,41]. At pH 6.5, the surface charge of the nanodrug was reversed to 2.61 ± 0.83, as a result of antibody release. Notably, a positive surface of the nanodrug will promote its cellular uptake. The aCD47 of the nanodrug was labeled with Alexa Fluor 488 and, when the pH was reduced, the fluorescence of the nanodrug solution at 520 nm gradually increased over time, apparently due to CDM cleavage-induced antibody release (Fig. 1E). Next, to mimic the tumor extracellular matrix and intracellular lysosomal environment, we explored *in vitro* drug release at pH 6.5 and 5.5. As shown in Fig. 1F, aCD47 was rarely released at pH 7.4, due to the high stability of the CDM linkage; however, on CDM cleavage, aCD47 release rapidly increased at pH 7.4, with over 70% released at 12 h. Ce6 was also released very slowly at pH 7.4, with less than 20% release at 24 h, and its release was only slightly increased at pH 6.5. In contrast, Ce6 release increased at pH 6.5 due to micelle disassembly (Fig. 1G). These *in vitro* drug release results demonstrate the dual pH sensitivity of Ce6@PPC-aCD47, to achieve the spatiotemporal release of aCD47 and Ce6 inside the tumor.

### Cell uptake, cytotoxicity, and PDT-induced ICD in vitro

**Figure 2:**
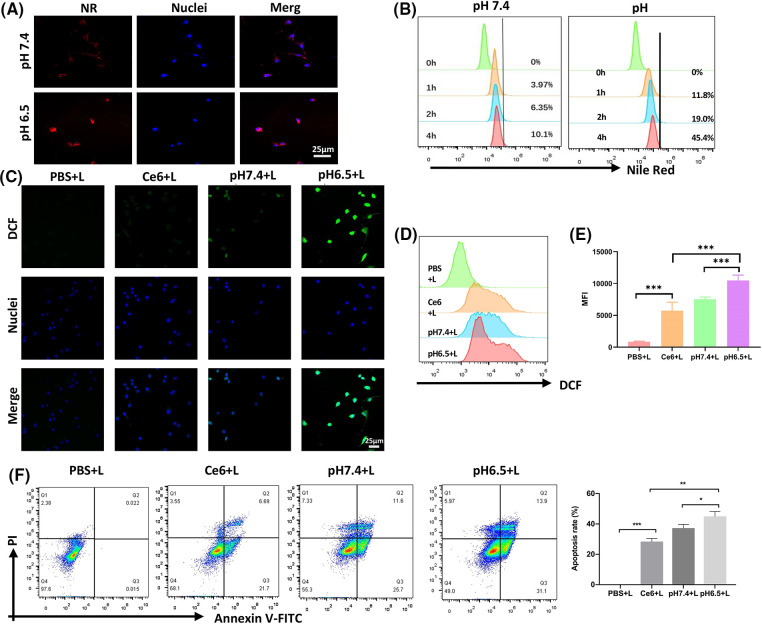
Nanodrug uptake and *in vitro* killing effects. Uptake of NR@PPC-aCD47 by K7M2 cells analyzed by confocal laser scanning microscopy (CLSM) (A) and flow cytometry, scale bar = 25 μm. (B). ROS in K7M2 cells treated with PBS, free Ce6, and Ce6@PPC-aCD47 analyzed by CLSM, scale bar = 25 μm. (C) and flow cytometry (D and E). (F) Apoptosis of K7M2 cells was tested by flow cytometer. *p < 0.05; **p < 0.01; ***p < 0.001.

CD47 can facilitate avoidance of ICD. CT26 cells with high CD47 expression were chosen to detect CD47 expression on K7M2 cells (Suppl. Fig. S3). To explore nanodrug uptake by the K7M2 osteosarcoma cell line, we used Nile Red, instead of Ce6, in the NR@PPC-aCD47 preparation, and conducted confocal microscopy and flow cytometry experiments. As shown in Fig. 2A, K7M2 cells were incubated with NR@PDPA-aCD47 at pH values of 7.4 and 6.5. Almost no NR@PPC-aCD47 was taken up by K7M2 cells at pH 7.4; however, internalization of the nanodrug into K7M2 cells increased significantly at pH 6.5, likely due to surface charge reversal [42]. Quantitative flow cytometry analysis also generated consistent results (Fig. 2B); K7M2 cells only displayed 10.1% particle uptake at pH 7.4, while uptake was 44.4% at pH 6.5. Nanodrug cytotoxicity was next determined by CCK-8 assay (Suppl. Fig. S4). Cells incubated with drug-free micelles showed no influence on K7M2 cell viability. In the absence of irradiation, the viability of K7M2 cells incubated with even 100 µg mL^−1^ of Ce6@PPC-aCD47 was >80%, indicating that the nanodrug exhibits low cytotoxicity; however, on irradiation, Ce6@PPC-aCD47 displayed significant concentration-dependent cytotoxic effects on K7M2 cells. The IC50 of Ce6 was 1 × 10^−6^ μM, likely due to ROS generation. Then flow cytometry assay was also used to detect the *in vitro* antitumor effects of the nanodrug. As shown in Fig. 2C, on irradiation, K7M2 cells incubated with Ce6@PPC-aCD47 at pH 6.5 displayed maximum cell apoptosis or necrosis, compared with same concentration of free Ce6 and Ce6@PPC-aCD47 at pH 7.4. These results suggest that Ce6@PPC-aCD47 can be effectively endocytosed by K7M2 cells under an acidic tumor microenvironment and enhance ROS generation, leading to higher levels of tumor cell apoptosis. Study also indicated ROS produced by PDT can trigger ICD and release damage-associated molecular patterns, such as CRT, HMGB1, and ATP [28]. Confocal laser scanning microscopy (CLSM) and flow cytometry assays were used to verify ROS generation by PDT. As shown in Fig. 2D, K7M2 cells incubated with Ce6@PPCaCD47 at pH 6.5 under laser exposure had significantly higher levels of ROS compared with cells incubated with Ce6@PDPA-aCD47 at pH 7.4 or Ce6. Consistent with these findings, K7M2 cells treated with Ce6@PPC-aCD47 at pH 6.5 with irradiation had higher mean fluorescence intensity than those subjected to any other treatments (Figs. 2E and 2F). During ICD, CRT translocates from the endoplasmic reticulum to the cytomembrane, which can facilitate DCs maturation and enhance macrophage phagocytosis. Therefore, we next investigated CRT levels via both CLSM observation and flow cytometry.

### PDT-mediated immunogenic cell death in K7M2 cells

**Figure 3:**
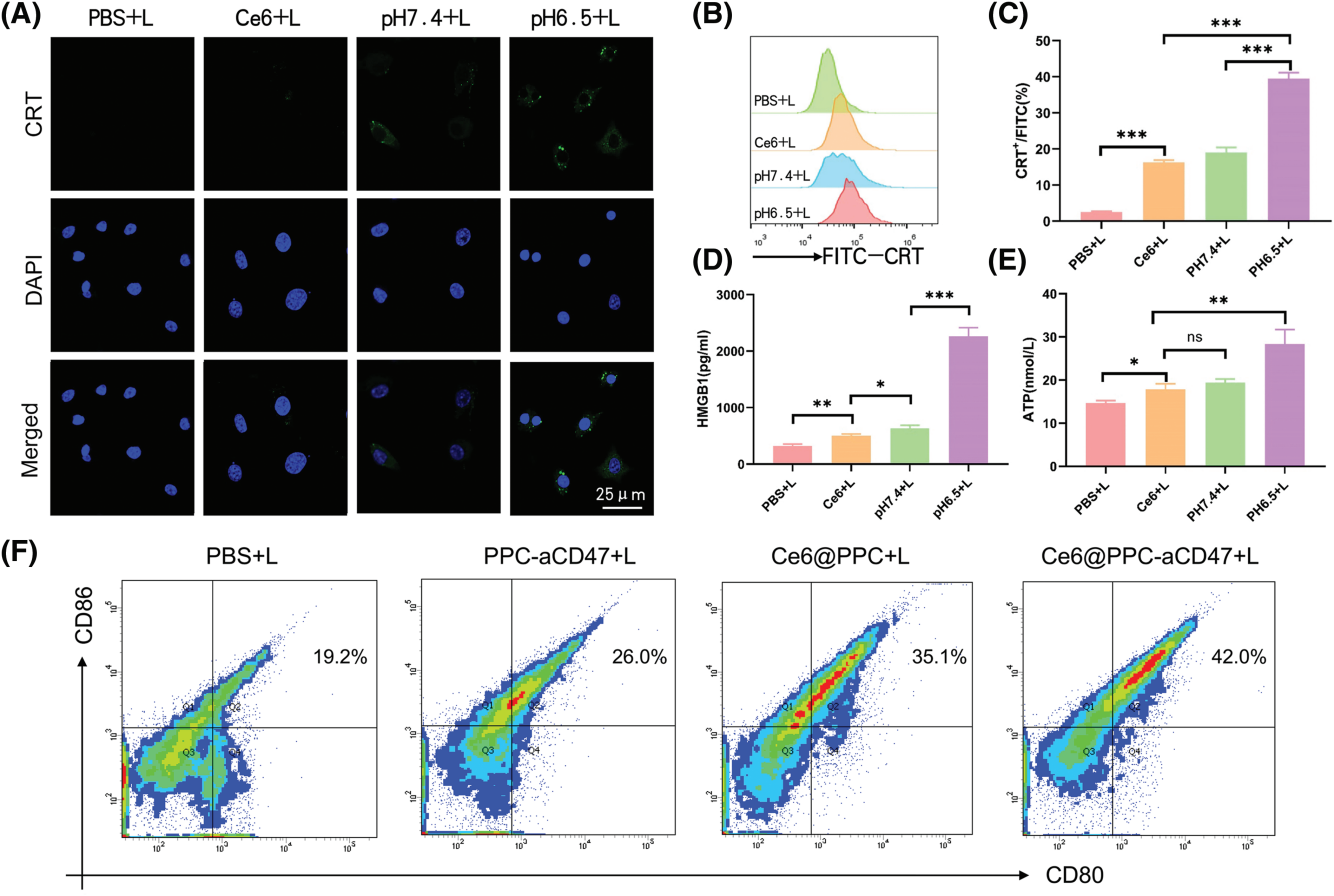
PDT-mediated immunogenic cell death in K7M2 cells. (A) Confocal imaging of CRT expression (green) on K7M2 cells, scale bar = 25 μm. (B) Flow cytometry analysis of CRT expression. (C) Statistical analysis of CRT expression. (D) The release of HMGB1. (E) ATP secretion. (F) Bone marrow dendritic cell maturation detected by flow cytometer. Ns, no statistical significance, *p < 0.05, **p < 0.01, ***p < 0.001.

Furthermore, our data also manifested that under irradiation, a strong CRT signal was observed in the Ce6@PPC-aCD47 (pH 6.5) group, and the CRT signal was much stronger in this group than those observed under other conditions (Fig. 3A). Similar results were revealed by flow cytometry, where cells incubated with Ce6@PPC-aCD47 at pH 6.5 had the highest CRT levels (Figs. 3B and 3C). HMGB1 and ATP release are also known as danger signals that can stimulate phagocytosis of dying tumor cells by DCs and macrophages. Therefore, ELISA analysis was used to measure HMGB1 and ATP secretion levels. Under irradiation, cells incubated with Ce6@PPC-aCD47 at pH 6.5 secreted more HMGB1 and ATP (Figs. 3D and 3E), similar to the findings for CRT. Immunofluorescence imaging to detect HMGB1 generated similar results (Suppl. Fig. S5). Next, we further investigated PDT- and aCD47-induced and antitumor immunity by detecting DC maturation. DCs from BALB/c mice were incubated with pretreated K7M2 cells and subsequent flow cytometry assay indicated that the percentage of mature DCs was reduced 24 h after incubation with K7M2 cells (Fig. 3F). The frequency of mature DCs increased when DCs were incubated with Ce6@PPC+L- or PPC-aCD47+L-pretreated K7M2 cells, while the Ce6@PPC-aCD47+L group showed an even higher level of DC maturation than that detected in the other groups.

### Tumor accumulation and biodistribution of Ce6@PPC-aCD47 in vivo

**Figure 4:**
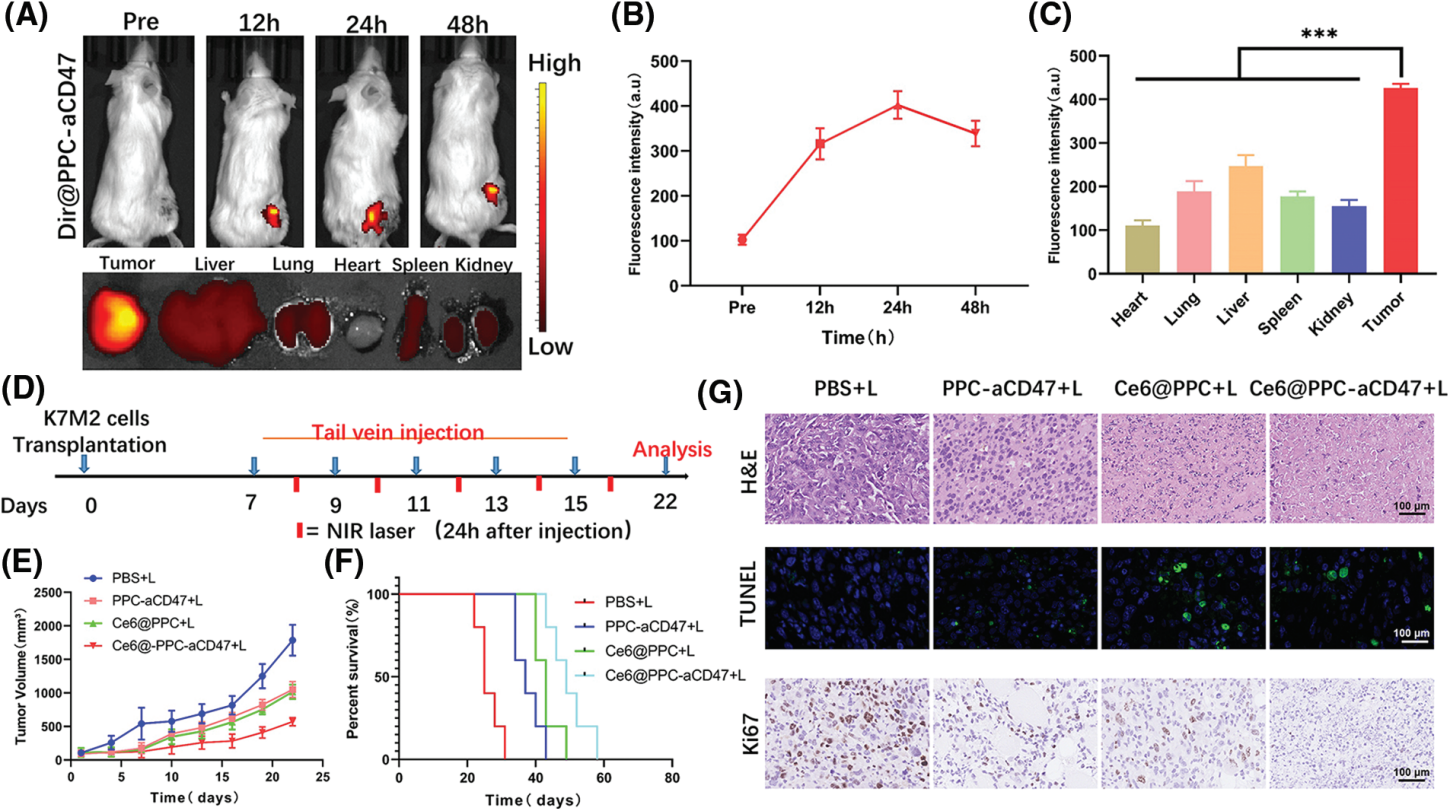
*In vivo* fluorescence imaging and synergistic antitumor effects of nanodrugs. (A) *In vivo* DiR fluorescence and *ex vivo* fluorescence 48 h after injection. (B) Quantitative analysis of fluorescence intensity values in tumors. (C) Fluorescence intensity values of main organs and tumors 48 h after injection. (D) Schematic illustration of therapeutic strategy. Tumor growth (E) and cumulative survival (F). (G) The pathological structure, apoptosis and Ki67 of the tissues were detected by H&E, TUNEL, and Ki67 staining, scale bar = 100 μm. ***p < 0.001.

Nanodrugs can accumulate in solid tumors due to tumor EPR effects [43,44]; therefore, we next investigated the accumulation and distribution of Ce6@PPC-aCD47 *In vivo*. As shown in Figs. 4A and 4B, DiR-labeled Ce6@PPC-aCD47 were i.v. injected to K7M2 tumor-bearing BALB/c mice. DiR intensity increased with time and peaked at 24 h, suggesting that this would be a good time point to achieve a better effect of PDT. Moreover, DiR intensity in the tumor site decreased slightly and remained strong at 48 h post-injection, indicating that Ce6@PPC-aCD47 can effectively and persistently accumulate in K7M2 tumors. At 48 h after injection, DiR-labeled Ce6@PPC-aCD47 had much higher fluorescence in tumors than in the heart, liver, spleen, lung, or kidney of experimental animals (Fig. 4C), indicating accumulation of the nanodrug in tumors, which is essential for targeted PDT treatment and activation of anti-tumor immune responses.

### Nanodrug antitumor efficacy in vivo

Some studies have demonstrated that aCD47 can inhibit the interaction of CD47 and SIRPα to facilitate DC maturation and tumor cell phagocytosis by TAMs [45,46]. Ce6-induced ICD of tumor cells can also release immunogenic signals to accelerate DC maturation for anti-tumor immunotherapy. To explore the synergistic potential of CE6@PPC-aCD47, a therapeutic schedule was carried out in model mice, as illustrated in Fig. 4D. Ce6-PPC+L or PPC-aCD47+L slightly suppressed tumor growth relative to PBS+L (Fig. 4E), while a much stronger inhibitory effect tumor growth was detected in mice receiving Ce6@PPC-aCD47+L, indicating a combined antitumor effect of exerted by codelivery of Ce6 and aCD47. Moreover, mice in the Ce6-PPC-aCD47+L group had the longest survival time (Fig. 4F). The inhibitory effect of the nanodrug on tumor growth was further investigated by H&E, Ki67, and TUNEL staining. Compared with PBS+L treatment, Ce6-PPC+L and PPC-aCD47+L resulted in remarkable cellular apoptosis and necrosis, suggesting the antitumor efficacy of both PDT and immunotherapy. Treatment with Ce6@PPC-aCD47+L further enhanced efficacy, indicating a synergistic effect of combined Ce6 and aCD47 (Fig. 4G). Ki67 immunohistochemistry was also used to evaluate cell proliferation, and Ce6@PPC-aCD47+L treatment again showed the highest efficacy in inhibiting tumor growth (Fig. 4G and Suppl. Fig. S6). To study the side effects of the nanodrug, we next conducted histological analyses of the major organs of the model mice and detected their hematological indices at the end of treatment. No obvious histological damage was observed in major organs (Suppl. Fig. S7). Furthermore, no differences in ALT, TBIL, or BUN were detected among mice subjected to different treatments (Suppl. Fig. S8). These findings indicate that the combined therapy strategy exhibited good biosafety and low side effects.

### Nanodrug-induced in vivo immune response

**Figure 5:**
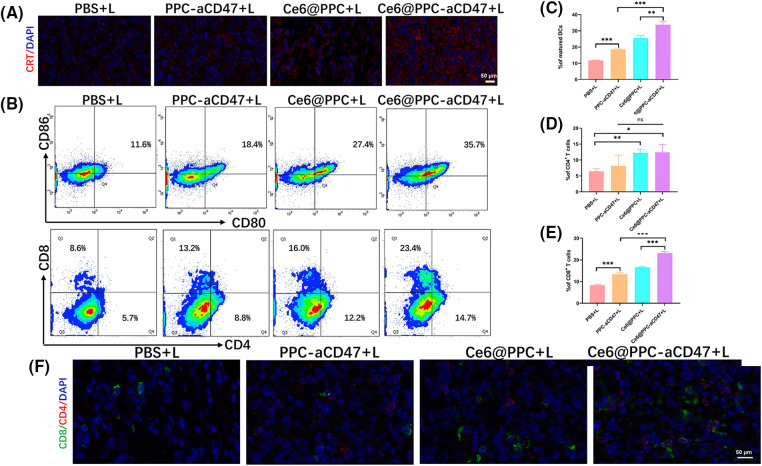
*In vivo* antitumor immune response. (A) CRT (red) in K7M2 cells. (B) Mature DCs in tumor tissues were analyzed by flow cytometer. CD8+ and CD4+ T cell ratios in tumor tissues. Statistical analysis of the ratio of mature DCs (C), CD4+ T cells (D), and CD4+ T cells (E). (F) CD4 and CD8 expressions, scale bar = 50 μm. *p < 0.05, **p < 0.01, ***p < 0.001.

To better understand the reasons for the much stronger effects of treatment with Ce6@PPC-aCD47+L than those of separate Ce6-PPC+L and PPC-aCD47+L treatments, tumor tissues from each group were collected at the end of treatment and related immune signals analyzed using histological and flow cytometry assays. Immunofluorescence staining of CRT was used to assess the level of ICD. Increased CRT was found in the Ce6-PPC+L and Ce6@PPC-aCD47+L treatment groups, indicating significant ICD induction by PDT of Ce6 (Fig. 5A). DC maturation is key for antigen presentation and further activation of effector T lymphocytes [47]. The percentage of mature DCs in tumors was examined by flow cytometry using mature DC biomarkers, CD80 and CD86. As shown in Figs. 5B and 5C, similar to CRT, the percentages of mature DCs in the Ce6-PPC+L and Ce6@PPC-aCD47+L-treated groups were much higher than those in the PBS+L treatment group. Intratumoral infiltration of effector T cells was also examined. Very little CD8+ T cell tumor infiltration was detected in the PBS+L group, while mice receiving other treatments displayed more CD8+ T cell infiltration of tumors (Figs. 5B and 5D). Specifically, the Ce6@PPC-aCD47+L group exhibited the most robust infiltration. CD4+ T cells can recognize tumor cells and interact with CD8+ T cells to suppress tumor growth. Flow cytometry analysis of CD4+ T cells showed similar results (Figs. 5B and 5E). Immunofluorescence staining of tumor sections also led to consistent results, demonstrating that Ce6@PPC-aCD47+L treatment led to the most remarkable infiltration of CD8+ T and CD4+ T cells (Fig. 5F). These findings suggest that blocking Ce6 and CD47 can effectively stimulate an adaptive immune response.

### Nanodrugs increased antitumor immune responses in mice receiving different treatments

**Figure 6:**
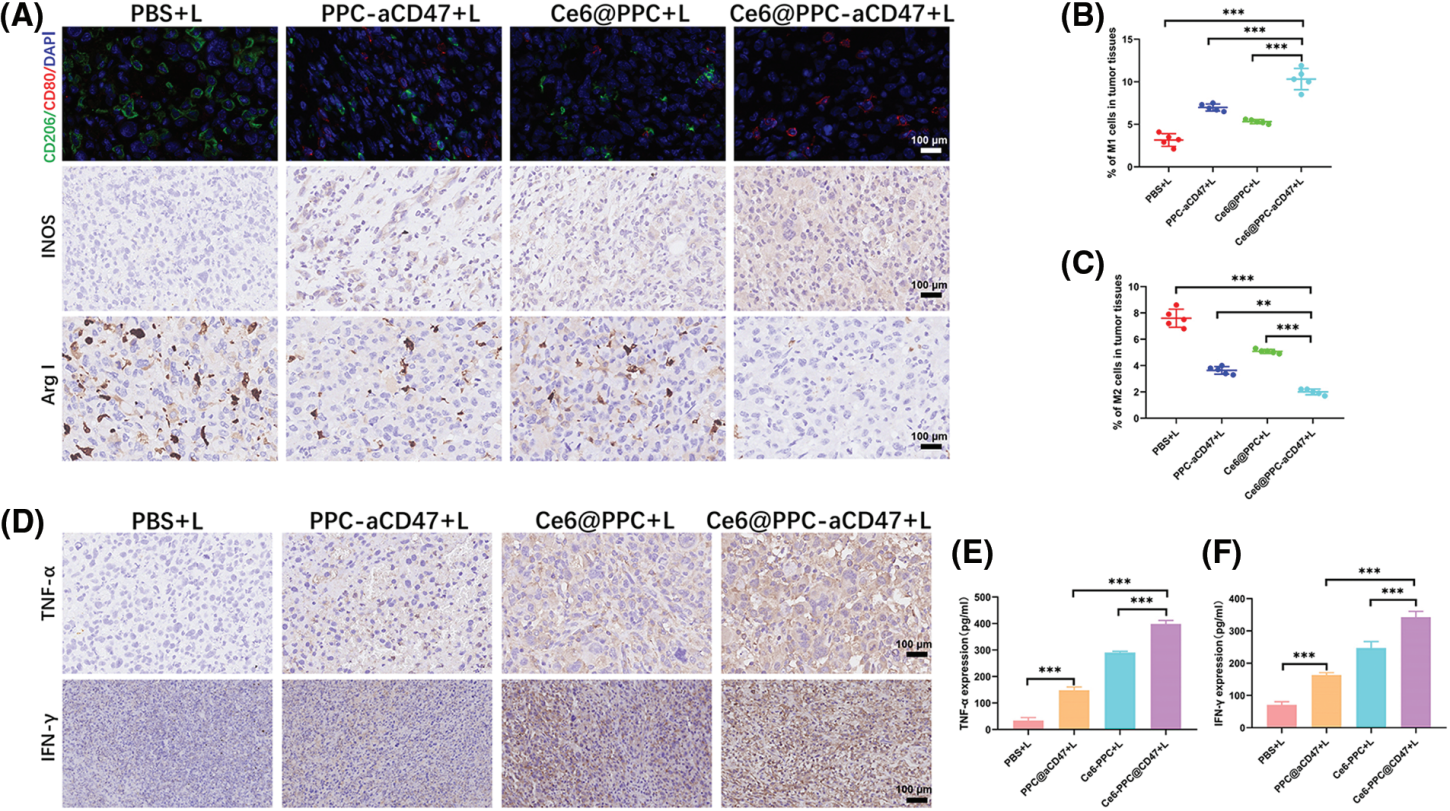
Nanodrugs enhanced antitumor immune responses in mice receiving different treatments. (A) Immunohistochemical and immunofluorescent staining of macrophage biomarkers (M1, CD80, INOS; M2, CD206, Arg-I), scale bar = 100 μm. (B) M1 phenotype macrophage (CD11b+F4/80+CD80+) ratios in tumor tissue. (C) M2 phenotype macrophage (CD11b+F4/80+CD206+) ratios in tumor tissue. (D) IFN-γ and TNF-α expressions were confirmed by IHC assay, scale bar = 100 μm. Levels of TNF-α (E) and IFN-γ (F) determined by ELISA. **p < 0.01, ***p < 0.001.

Both surface CRT expression during ICD and CD47 blockage can also promote the activation of TAMs leading to antitumor effects [48,49]. The IHC and immunofluorescent staining demonstrated decreased M2-like TAMs and increased M1-like TAMs in the three nanodrug treatment groups (Fig. 6A and Suppl. Fig. S6). Then we assessed the ratio of pro-tumoral M2-like phenotype and antitumoral M1-like phenotype TAMs. M1-like TAM infiltration in tumor tissues from the Ce6@PPC-aCD47+L treatment group was higher than that than in the other groups (Suppl. Fig. S9 and Fig. 6B). In contrast, significantly fewer M2-like TAMs were detected in tumors from the Ce6-PPC+L and PPC-aCD47+L treatment groups, relative to controls, while Ce6@PPC-aCD47+L treatment further reduced M2-like TAM infiltration (Suppl. Fig. S10 and Fig. 6C). Moreover, IHC staining generated consistent results (Fig. 6D and Suppl. Fig. S6). Further, we detected IFN-γ and TNF-α, to evaluate the immunotherapeutic effect of the nanodrug. Compared to the PBS+L group, the Ce6-PPC+L, and PPC-aCD47+L treatment groups showed increased IFN-γ and TNF-α secretion, and the Ce6@PPC-aCD47+L group had even higher secretion levels (Figs. 6E and 6F). The enhanced antitumor immune response was clearly due to the synergistic effect of Ce6 and aCD47. To study the effect of the nanodrug on immune memory, effector memory T cells (TEM) in the spleens of model mice were evaluated by flow cytometry. The TEM percentage was significantly elevated in Ce6@PPC-aCD47+L-treated mice relative to those in the other treatment groups (Suppl. Fig. S11). These results suggest that Ce6 and aCD47 may function together to prevent osteosarcoma recurrence and metastasis.

## Discussion

Convincing evidence from both basic experimental studies and clinical research supports a bright future for inhibition of immune checkpoints in treating cancer [50]. Determining how to improve the immunosuppressive environment will be key to improving immunotherapy efficacy. Many studies have demonstrated that PDT can both generate ROS, to directly kill cancer cells, and induce ICD. Molecules, such as CRT, ATP, and HMGBP, released from dying tumor cells, promote DC maturation to stimulate T cell-mediated cancer cell killing and promote phagocytosis by TAMs [17,31].

Nanocarrier-encapsulated drugs can improve the biodistribution of drugs *in vivo* and target them to tumors through the EPR effect, thus reducing toxicity and achieving cancer therapeutic effects. Currently, nanodrug carriers mainly include polymer micelles [51], polymer vesicles [52], nanogels [53], and liposomes [54]. It has been demonstrated that stimuli-responsive release of drugs at the tumor site facilitates the tumor suppression effect. Among them, pH-responsive drug release is one of the proven strategies. The pH in human tumor tissue environment is lower than that of neutral normal tissue, while the pH of lysosomes and endosomes in tumor cells is even lower, 4.5–5.0 and 5.0–6.5, respectively. The design of nano-drug carriers with pH-responsive drug release function using the above pH variation is a hot research topic in the field of chemotherapeutic drug delivery in recent years [55,56]. Several studies have indicated that there are high levels of CD47 in osteosarcoma cells and that osteosarcomas can be inhibited by aCD47 [20]. Combination therapy using PDT and aCD47 has achieved success in other cancers [31], but its effectiveness in osteosarcoma is unclear and its influence on macrophages has not been evaluated. Here, we designed a core-shell nanodrug with dual pH sensitivity to carry aCD47 on its surface and encapsulate Ce6 in its core, and increase drug accumulation via a tumor EPR effect. In tumors, the nanodrug may release aCD47 due to the acidic microenvironment (pH approximately 6.5). The nanodrug also released CUR in lysosomes (pH approximately 5.5). Under irradiation, Ce6 taken up by tumor cells could induce ROS to kill the tumor cells, and promote immune response activation to enhance the therapeutic effect of aCD47. These findings demonstrate a strong synergistic effect of PDT and aCD47 in osteosarcomas, which significantly suppressed tumor growth and prolonged survival time in model mice. To our knowledge, this is the first study to assess the effects of combination PDT and immunotherapy using nanomicelles to strengthen the therapeutic effect and reduce side effects.

PDT refers to the use of appropriate wavelength light irradiation and activation of photosensitizer molecules located in the tumor tissue or cells [57]. The activation of photosensitizer molecules can produce a variety of ROS in the local tumor tissues. ROS can oxidize cells and kill tumor cells through necrosis, apoptosis, autophagy, tumor-specific immunity and other mechanisms [58,59]. PDT has emerged as a new alternative intervention for anti-cancer therapy. CD47 is an extracellular ligand of SIRPα, and the combination of SIRPα and CD47 reduces the phagocytic activity of macrophages by producing inhibitory signals, resulting in tumor immune escape [60]. CD47 is highly expressed in multiple malignant tumors [61–63]. Application of aCD47 can specifically act on tumor cells to promote the phagocytosis of macrophages [64,65]. Previous investigators also demonstrated that the ICD induced by PDT and other therapeutic methods can significantly improve the effects of immunotherapy [37,47]. However, a combination of doxorubicin and PD-L1 inhibitors was only slightly more effective than PD-L1 alone for osteosarcoma treatment [66]. In our study, the ICD caused by PDT plus aCD47 enhanced DC maturation and macrophage activation, and local irradiation of tumors could reduce systemic side effects caused by other therapies, such as chemotherapy.

However, this study also has some limitations. For instance, more osteosarcoma cell lines should be applied to verify the immune-activating and anti-tumor effects of nanodrug of codelivery of aCD47 and Ce6 using a dual pH-sensitive nanodrug *in vitro*.

## Conclusions

We describe preparation of a dual-pH sensitive micelle incorporating the photosensitizer, Ce6, in its core and conjugated with aCD47 for application in synergistic PDT and immunotherapy of osteosarcoma. The prepared nanodrug showed excellent biocompatibility and could regulate Ce6 and aCD47 release in response to an acidic tumor microenvironment and lysosomal acidity in cancer cells. Ce6 delivered to tumors could exert PDT under irradiation, thereby promoting the generation of tumor-associated antigens. Further, aCD47 could stimulate phagocytosis by TAMs and DCs, as well as T cell activation. *In vivo* experiments demonstrated that Ce6@PPC-aCD47 treatment under irradiation induced the most significant inhibition of K7M2 tumor growth. Therefore, combination therapy with Ce6-mediated PDT and CD47 blockage may serve as a novel strategy for osteosarcoma treatment.

## Supplementary Materials

Figure S1 Copolymer synthesis [39].

Figure S2 Stability of Ce6@PPC-aCD47 in PBS containing 10% FBS, detected by dynamic lighting scattering (n = 3).

Figure S3 (A) CD47 expression on CT26 colorectal and K7M2 osteosarcoma cells. (B) Quantitative analysis of CD47 surface expression on CT26 and K7M2 tumor cells. (C) CD47 expression in K7M2 cells. Sections were stained with DAPI and FITC, scale bar = 50 μm.

Figure S4 Viability of K7M2 cells incubated with blank micelle (A) and Ce6@PPC-aCD47 without (B) or with (C) irradiation.

Figure S5 Confocal laser scanning microscopy examination of HMGB1 secretion in K7M2 cells incubated with PBS, free Ce6, and Ce6@PPC-aCD47 at pH7.4 and pH6.5 under irradiation, scale bar = 25 μm.

Figure S6 Quantitative results of IHC assay. (A) Ki67, (B) INOS and Arg I (C) TNF-α and IFN-γ.

Figure S7 Hematoxylin and eosin staining of heart, liver, spleen, lung, and kidney samples, scale bar = 25 μm.

Figure S8 Hematological indices (n = 3, mean ± SD). Ns, no statistical significance.

Figure S9 Flow cytometric analysis of M1 macrophages (F4/80+CD11b+CD80+).

Figure S10 Flow cytometric analysis of M2 macrophages (F4/80+CD11b+CD206+).

Figure S11 Flow cytometric analysis of effector memory T cells in spleens (gated on CD3+CD8+CD44+ CD66L-).

## Data Availability

The datasets used and/or analyzed during the current study are available from the corresponding author on reasonable request.

## Supplementary Materials

The supplementary material is available online at https://doi.org/10.32604/or.2023.030767.
