# Supplementary figures and images for: Codelivery of anti-CD47 antibody and chlorin e6 using a dual pH-sensitive nanodrug for photodynamic immunotherapy of osteosarcoma
Source: Oncol Res. 2024 Mar 20;32(4):691–702. doi: 10.32604/or.2023.030767 (PMC10972781; doi:10.32604/or.2023.030767)

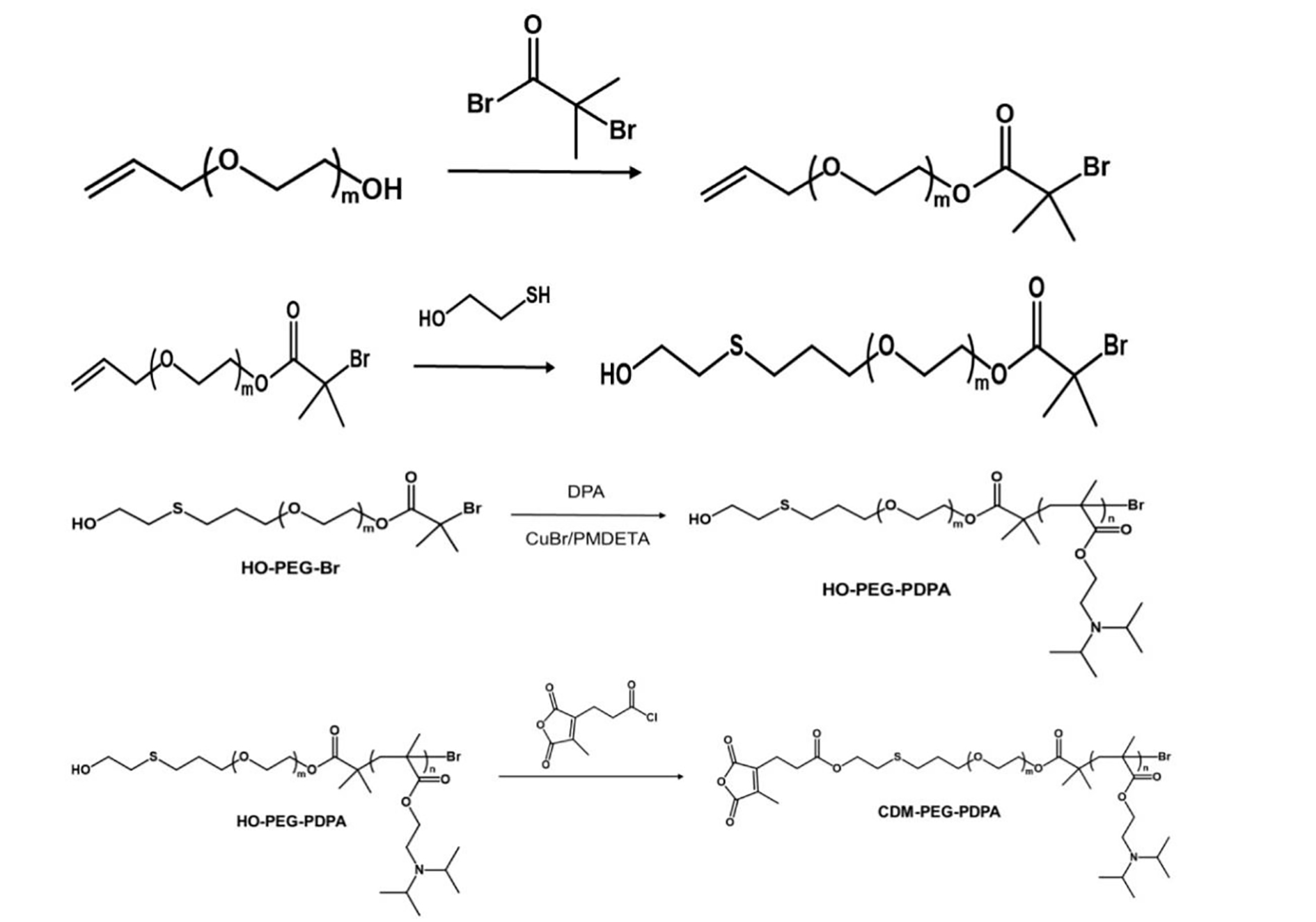

Supplement: Figure S1 [file OncolRes-32-30767-s001.tif]

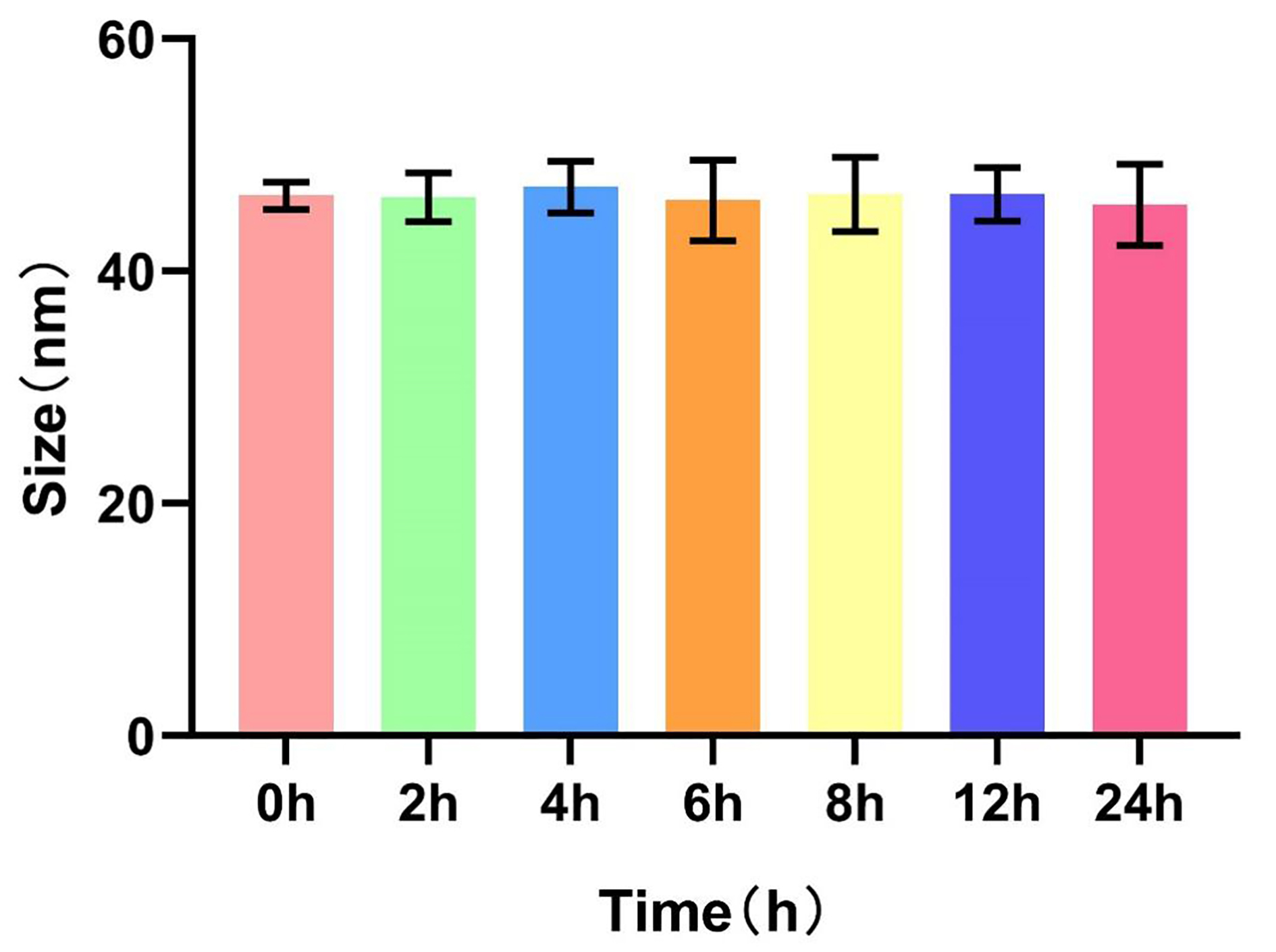

Supplement: Figure S2 [file OncolRes-32-30767-s002.tif]

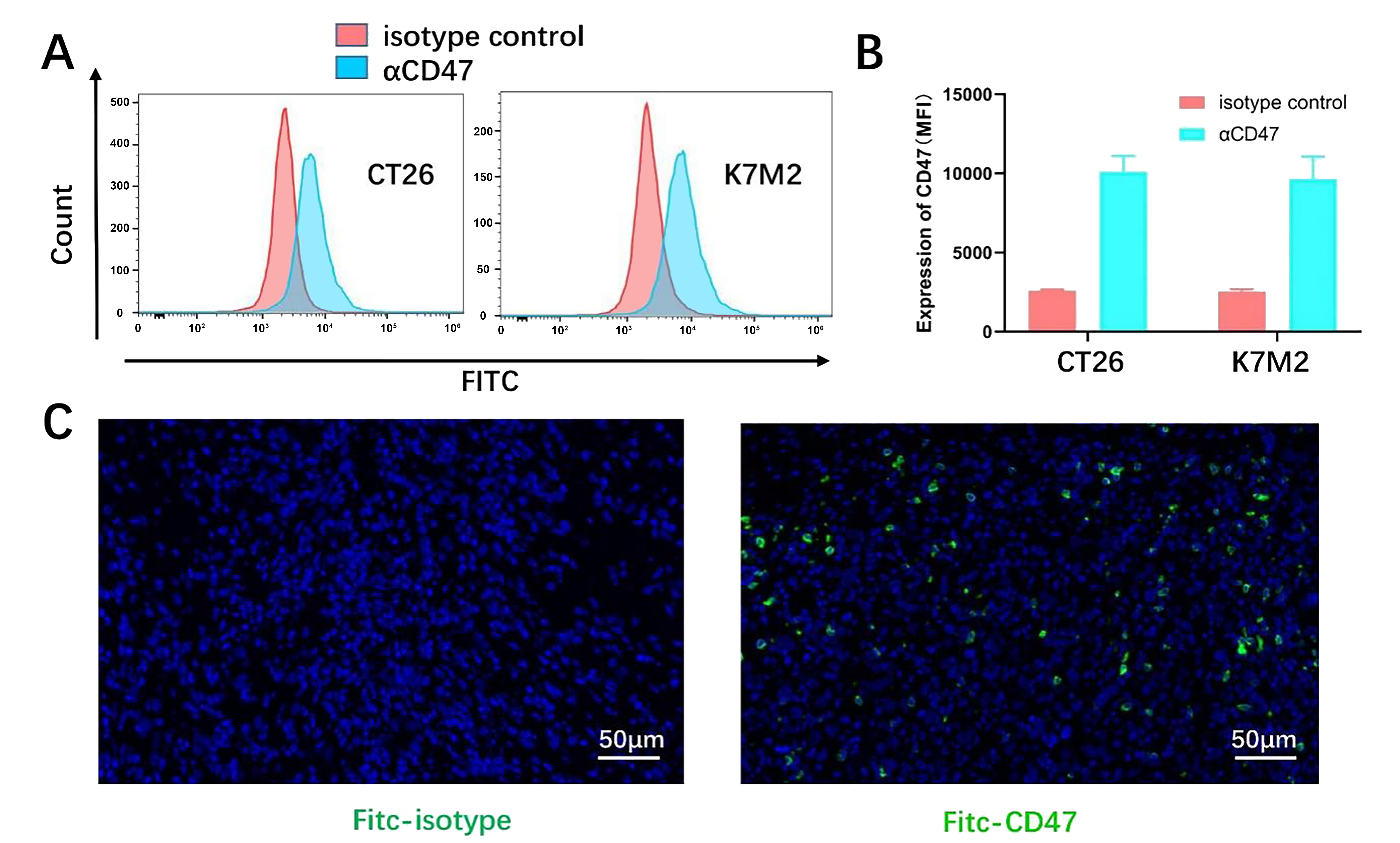

Supplement: Figure S3 [file OncolRes-32-30767-s003.tif]

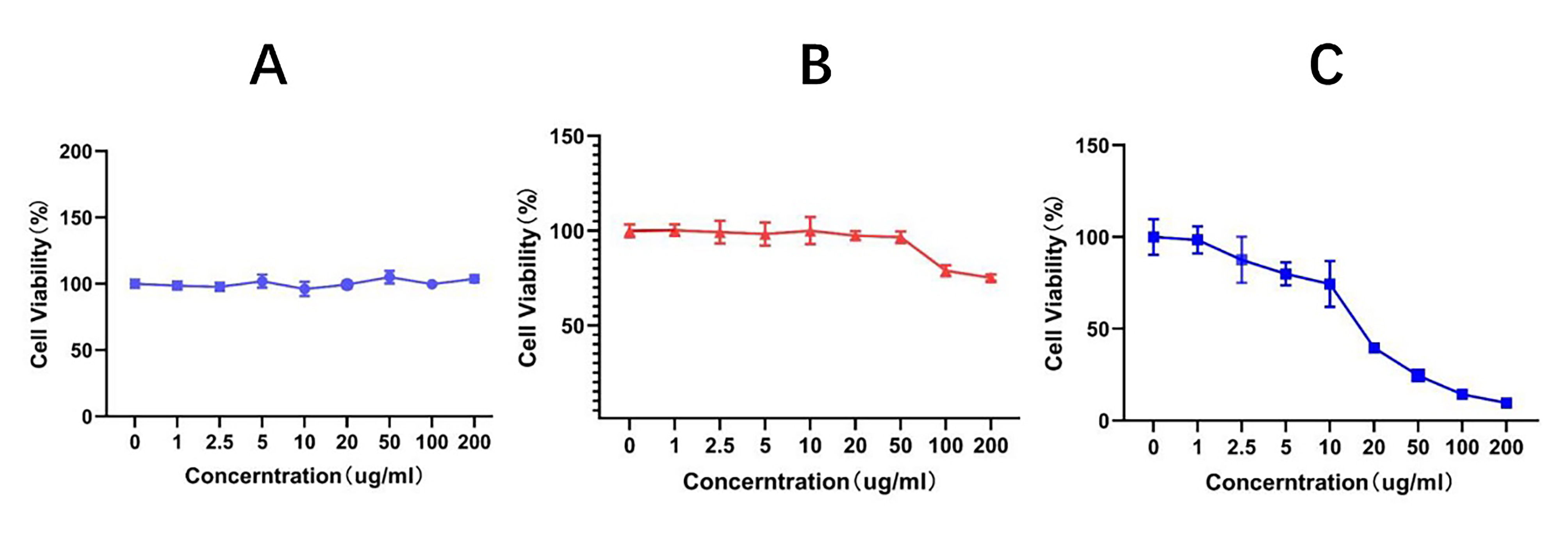

Supplement: Figure S4 [file OncolRes-32-30767-s004.tif]

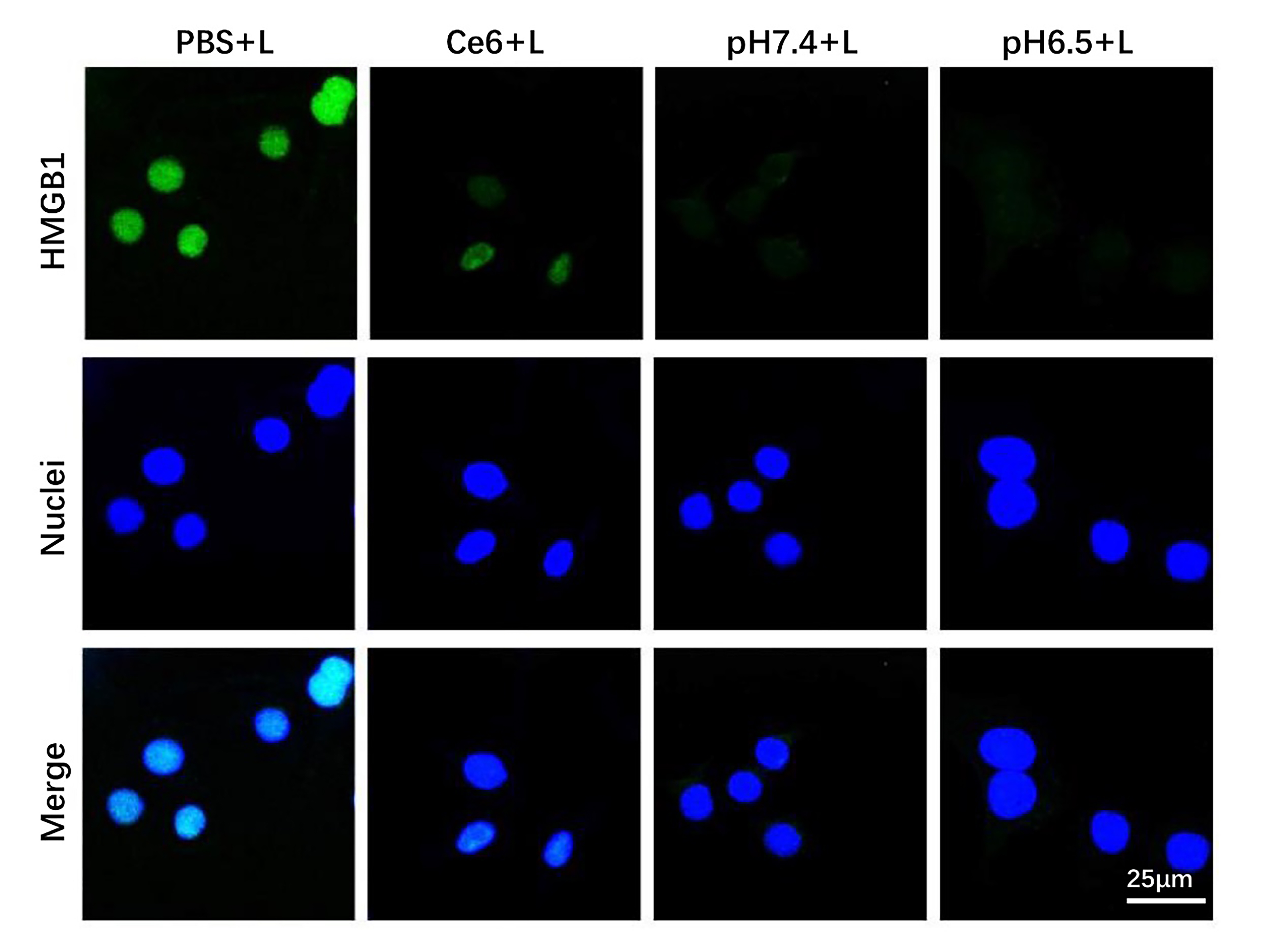

Supplement: Figure S5 [file OncolRes-32-30767-s005.tif]

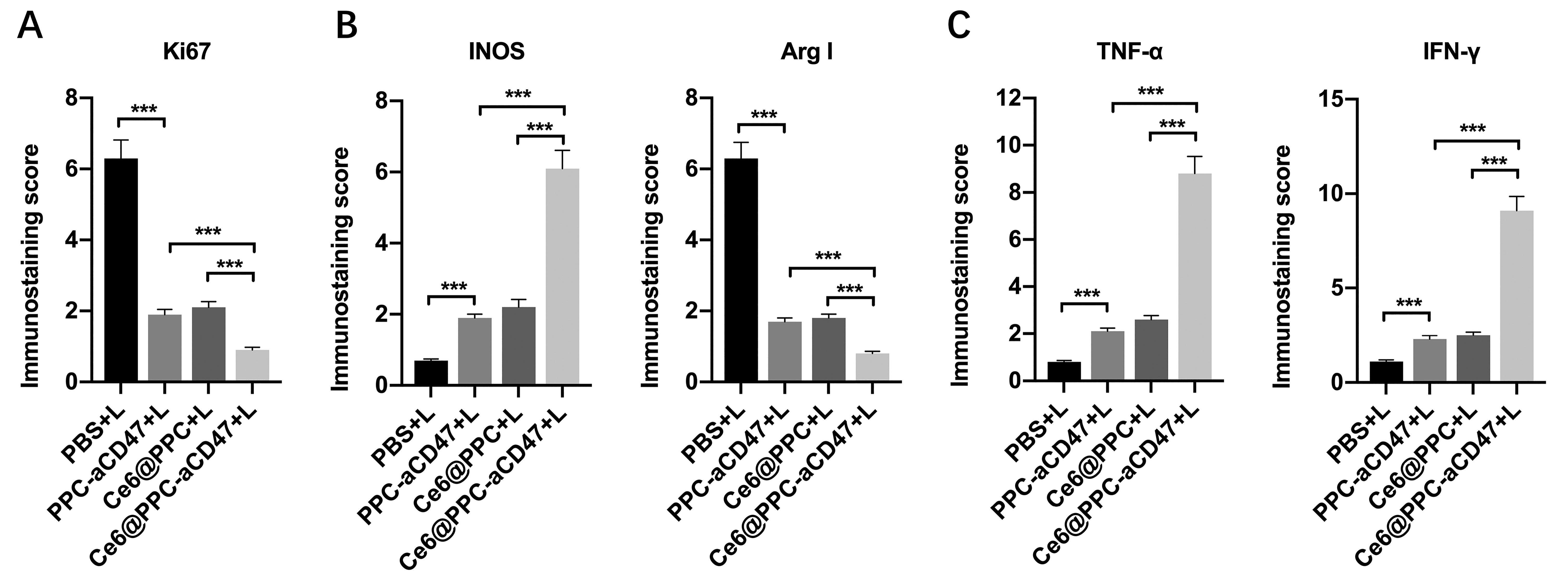

Supplement: Figure S6 [file OncolRes-32-30767-s006.tif]

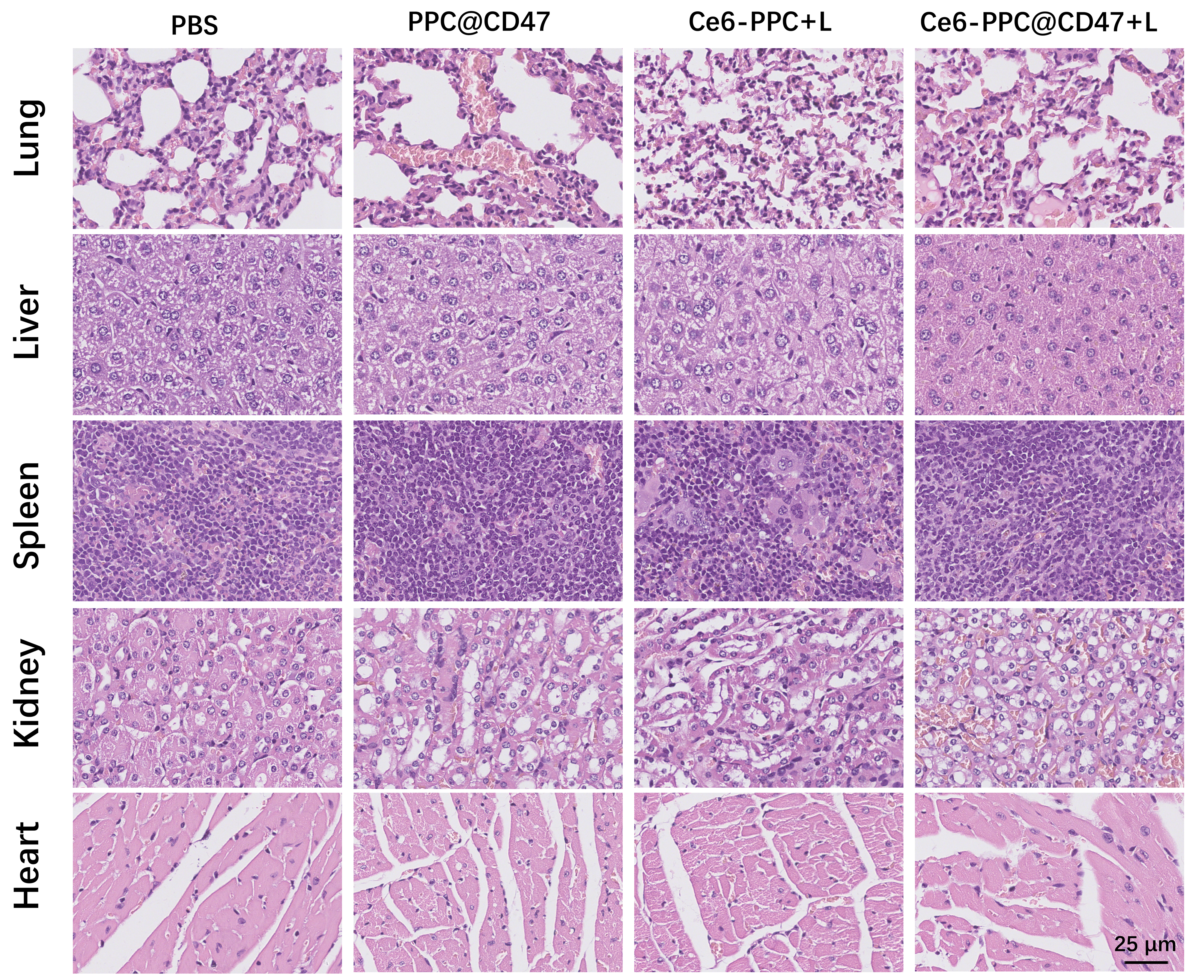

Supplement: Figure S7 [file OncolRes-32-30767-s007.tif]

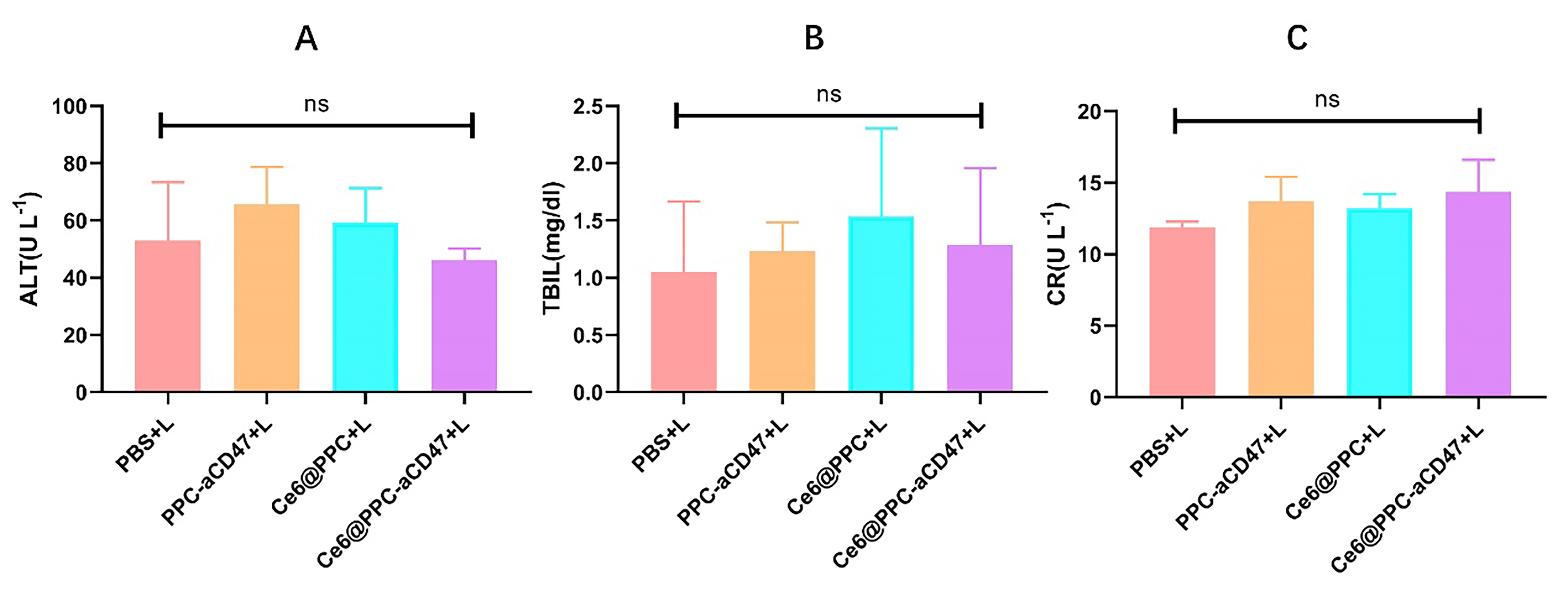

Supplement: Figure S8 [file OncolRes-32-30767-s008.tif]

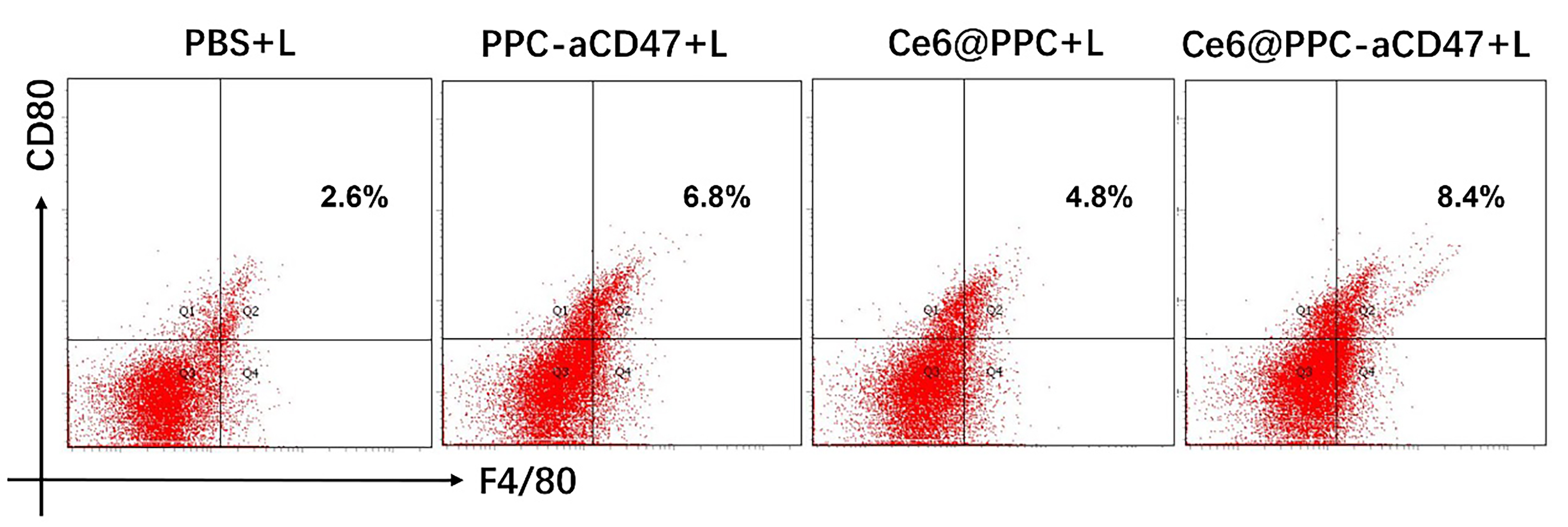

Supplement: Figure S9 [file OncolRes-32-30767-s009.tif]

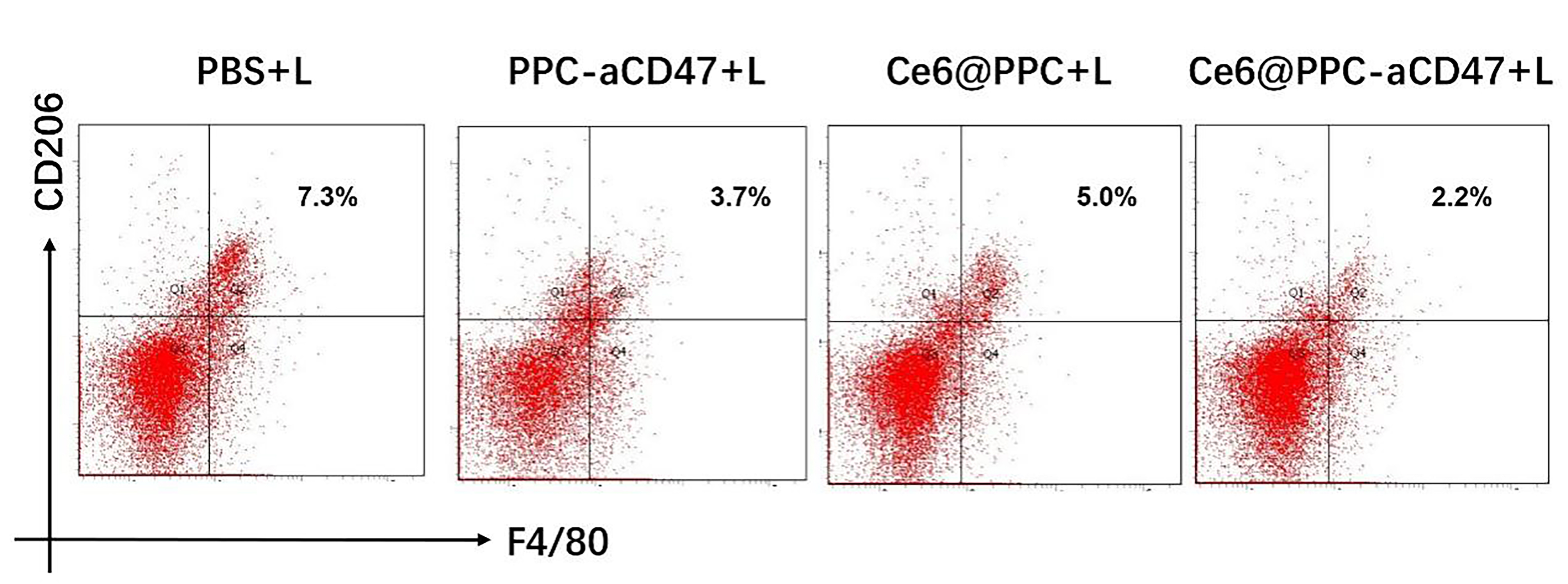

Supplement: Figure S10 [file OncolRes-32-30767-s010.tif]

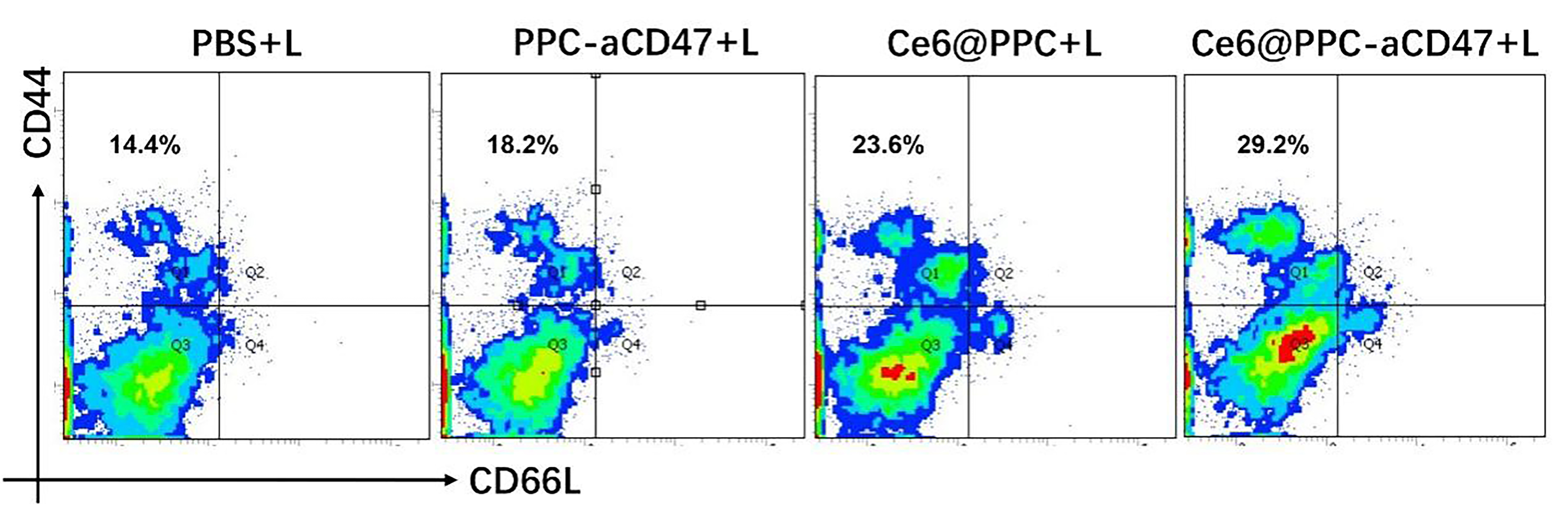

Supplement: Figure S11 [file OncolRes-32-30767-s011.tif]
